# Variables Related to Working Capability among Swiss Patients with Multiple Sclerosis—A Cohort Study

**DOI:** 10.1371/journal.pone.0121856

**Published:** 2015-04-13

**Authors:** Oliver Findling, Magdalena Baltisberger, Simon Jung, Christian P. Kamm, Heinrich P. Mattle, Johann Sellner

**Affiliations:** 1 Department of Neurology, Inselspital, University Hospital Bern, University of Bern, Bern, Switzerland; 2 Department of Neurology, Christian-Doppler-Klinik, Paracelsus Medizinische Universität, Salzburg, Austria; University Hospital Basel, SWITZERLAND

## Abstract

**Introduction:**

Reduced working capability is one of the most devastating consequences of multiple sclerosis (MS). We aimed to study working capability and related variables in Swiss MS patients.

**Materials and Methods:**

A cross-sectional analysis of employment status and risk factors for reduced working capability among MS patients treated at our outpatient clinic. A questionnaire was mailed to 644 MS patients and returned by 69.7%. 405 patients (66% female, mean age 44.2 years (SD±10.2), median EDSS 3.0 (SD±1.8)) were eligible for subsequent analysis.

**Results:**

After a mean disease duration of 12.3 years (SD±8.25), full or part time employment was declared by 26.7% and 25.7%, respectively. Incapacity to work was reported by 27.1%. A total of 52.8% specified MS as the cause for altered working capability, whereas 20.5% cited reasons unrelated to the disorder. Even with minimal disability (EDSS<3) a significant proportion of patients (24%) reported reduced working capability. Among the MS-specific restricting factors were fatigue (47.6%), sensorimotor deficits (31.1%), impaired vision (3.3%) and pain (2.8%).

**Conclusion:**

MS continues to takes its toll on the professional life of the patients early in the course. While complete incapacity becomes relevant with moderate to severe disability, many patients scale down to part-time even with minimal impairment.

## Introduction

Loss of working capability is a significant concern among individuals with multiple sclerosis (MS) and bears profound consequences including financial, social, and mental health implications. Notably, surveys from Australia, Western Europe and the United States revealed that up to 40% of all MS patients in the working age are currently unable to work [1,2]. In Switzerland, however, a country with nearly full employment in the general population, Kobelt and co-workers identified even lower employment rates (34.7%) [3]. Among the explanations were a sampling bias towards more advanced disease and lower employment rate [3]. Additional contributing factors might be related to the coverage by the social security system and early retirement options for chronic disabled patients in the country [4]. Being unable to work is furthermore associated with impaired health-related quality of life. Patti and co-workers reported that both occupation and educational levels were significant and independent predictors for the latter [5]. The life of patients with MS is disrupted at a phase of life when they are highly productive and forward-looking, as the disorder manifests most commonly between 20 and 40 years of age [6]. Indeed, being unable to work brings stress not only to the patient but also the caregivers, and requires re-evaluating of life goals defined through professional achievement [7].

Key variables related to an altered vocational status have been identified. These include disease progression, older age at diagnosis, and hard physical work [1]. Moreover, the lack of flexible working hours, the inability to have flexible resting times at work, a lack of understanding from colleagues and employers as well as the personal attitude were identified as main non-disease-specific reasons for early retirement [8–11]. Less consistent risk factors are gender (males more likely to remain employed), cognitive deficits, and longer time since diagnosis [12]. While depression has been consistently related to employment status in general population samples, this was not the case in MS [13,14].

The aim of the present study was to assess the employment status in a real life setting in patients treated at our MS outpatient clinic. Furthermore, we aimed to identify demographic and potentially modifiable risk factors for the loss of working capability.

## Materials and Methods

### Ethics Statement

The study was approved by the local ethical committee (permit number 15-08-2007) of the Bern University Hospital. A cross-sectional postal questionnaire-based survey among the MS patients who were treated at our MS centre during 2009 was conducted.

### Survey

The MS outpatient clinic at the University Hospital Berne/Switzerland has a catchment area of around 1.5 million people and provides diagnostic services and comprehensive care for MS patients at all stages of the disease with more than thousand patient contacts per year. A cross-sectional postal questionnaire-based survey among the MS patients who were treated at our MS centre during 2009 was conducted. We set up a multiple-choice questionnaire which made reference to the current employment and education system in Switzerland, current working capability and educational background. Working capability was grouped into: i) full time, ii) part-time and iii) complete inability to work. In case of reduced working capability we asked for the point in time and the reason for scaling down working capability. Educational background was assessed by the highest professional education level. The degree of education was grouped as follows: i) no vocational qualification, ii) industrial/off-the-job-training, iii) polytechnic degree, and iv) University degree. Higher educational background referred to University or polytechnic degree. The questionnaire discriminated between unemployment due to labour market reasons and partly or full inability to work due to disease related reasons as well as not working due to other reasons like childcare, household etc. The questionnaire was tested for reliability and consistency with a group of healthy persons of different age and was supplemented by data derived from the most recent medical report.

### Participants

Questionnaires were sent to all patients that were treated at our MS outpatient clinic during the last 12 months (n = 644, 66.1% female) and returned by 449 (69.7%). We restricted the time till return of the questionnaire to 8 weeks. Individuals above the national retirement age (women 64 years, men 65 years) (n = 33) or incomplete patient records (n = 11) were excluded. A total of 405 surveys (66% women) were subjected to further analysis. The cohort had a mean age of 44.2 years (SD ±10.2, range 18–65), median EDSS of 3.0 (SD ± 1.8, range 0–8.5) and mean disease duration of 12.32 years (SD ± 8.25, range 1–43).

### Disability groups

According to the pivotal study by C. Polman and R. Rudick we formed 3 groups of considering the degree of disability by using the Expanded Disability Status Scale (EDSS) [15,16]. The group of minimally disabled consisted of patients with an EDSS between 0 and 2.5. Patients with an EDSS from 3.0 to 4.5 where considered as moderately disabled and patients with an EDSS of 5.0 and higher as severely disabled [15,16].

### Statistical analysis

Statistical analysis was performed with IBM SPSS Statistics 21 (IBM Corporation 1989, 2012). Group comparisons were made based on employment status and disability and were undertaken for demographic and clinical variables by using Kruskal-Wallis-Test and Mann-Whitney-Test. Chi-squared test was used to calculate the distribution of gender and level of education in relation to the working capability. We used a multiple logistic regression model to estimate the association (odds ratio (OR)) between employment status and disability (EDSS), gender, disease course, age, disease duration and level of education. Significance was accepted at a level of <0.05.

## Results

Fulltime employment was declared by 108 patients (26.7%). In contrast, complete inability to work was reported by 110 patients (27.1%) and 104 patients (25.7%) had scaled down to part-time because of disease-related inability to pursue full-time work. Four of these patients stated that they are unemployed because they are not able to find a job adapted to their disability. The median time between first symptoms and scaling down the working capability was 6.59 years (±7.55). Eighty-three patients (20.5%) reported other reasons than MS for altered work, mostly related to care for children and household. Among the cohort of non-MS-related causes most were women (90.4%). Most of them had part time work (n = 56) and 4 were considered as unemployed due to labour market reasons. Except gender the demographic data of this group were not significantly different from those who work fulltime. For further analysis of risk factors influencing working capability these patients were excluded.

Patients who were retired part-time or full had significantly higher EDSS, age and disease duration compared to those who worked fulltime. Women were significantly underweighted in the group of fulltime-employed patients. Men and patients with higher education level were more likely fulltime employed. The portion of patients treated with a disease modifying treatment (DMT) was higher in the part-time and not working patients (p = 0.04). However, in treated patients the timespan between first symptoms and treatment initiation was significantly longer in fulltime-retired patients compared to fulltime-employed patients. The detailed data are shown in Table 1.

**Table 1:** Demographic data of all patients (N = 405) in relation to working capability.

	Fulltime working	Part-time working		Fulltime retired		Reduced work due to other reasons
**N (%)**	108 (26.7)	104 (25.7)		110 (27.1)		83 (20.5)
**Female (%)**	58 (53.7%)	68 (65.4%)	*p<0*.*001* *	73 (66.4%)	*p<0*.*001* *	75 (90.4%)
**Mean age (SD)**	40.9 (±9.6)	44.9 (±9.0)	*p = 0*.*02* *	48.2 (±10.3)	*p<0*.*001* * *p = 0*.*021* **	41.9 (±10.2)
**Mean disease duration (SD)**	10.4 (±8.1)	12.4 (±7.7)	*p = 0*.*014* *	16.0 (±9.1)	*p<0*.*001* * *p = 0*.*004* **	9.8 (±6.2)
**Median EDSS (SD)**	2.0 (±1.2)	3.0 (±1.4)	*p<0*.*001* *	5.0 (±1.6)	*p<0*.*001* * *p<0*.*001* **	2.0 (±1.3)
**Disease course, N (%)**						
**CIS**	0 (0)	2 (1.9)		0 (0)		2 (2.4)
**RRMS**	96 (88.9)	79 (76.0)		60 (54.5)		72 (86.8)
**SPMS**	8 (7.4)	20 (19.2)		45 (41.0)		5 (6.0)
**PPMS**	4 (3.7)	3 (2.9)		5 (4.5)		4 (4.8)
**Treatment with DMT**						
**N (%)**	82 (75.9)	87 (83.7)	*p = 0*.*04* *	96 (87.3)	*p = 0*.*04* *	61 (73.5)
**Mean time to treatment initiation (SD)**	5.01 (7.6)	6.2 (7.1)		8.1 (8.4)	*p = 0*.*001* * *p = 0*.*05* **	
**Education level, N (%)**						
**University/ polytechnic**	25 (23.1)	20 (19.2)		13 (11.9)	*p = 0*.*008* *	20 (24.1)
**Off-the-job/ industrial training**	76 (70.4)	80 (76.9)		75 (69.1)		55 (66.3)
**No vocational education**	7 (6.5)	4 (3.9)		22 (20.0)		8 (9.6)

*Compared to fulltime working

**Compared to part-time working

After applying a multiple logistic regression model EDSS, gender and education level remained as independent risk factors for impaired working capability. These data are shown in Table 2.

**Table 2:** Odds ratios (OR) for factors associated with working capability compared to fulltime working patients.

Risk factor	Part-time working		Fulltime retired	
	p-value	OR (95%CI)	p-value	OR (95%CI)
**EDSS**	<0.001	2.1 (1.6–2.9)	<0.001	3.9 (2.8–5.4)
**Female sex**	<0.001	3.4 (1.7–6.6)	0.001	3.9 (1.8–8.7)
**Age**	0.06	1.0 (1.0–1.1)	0.03	1.1 (1.0–1.1)
**Disease duration**	0.56	1.0 (0.9–1.1)	0.80	1.0 (0.9–1.1)
**Higher education level**	0.015	6.8 (1.4–32.4)	0.045	5.2 (1.0–25.9)
Time to treatment*	0.47	1.0 (0.9–1.1)	0.20	1.0 (0.8–1.1)

*Analysed in treated patients only (n = 264)

**Fig 1:**
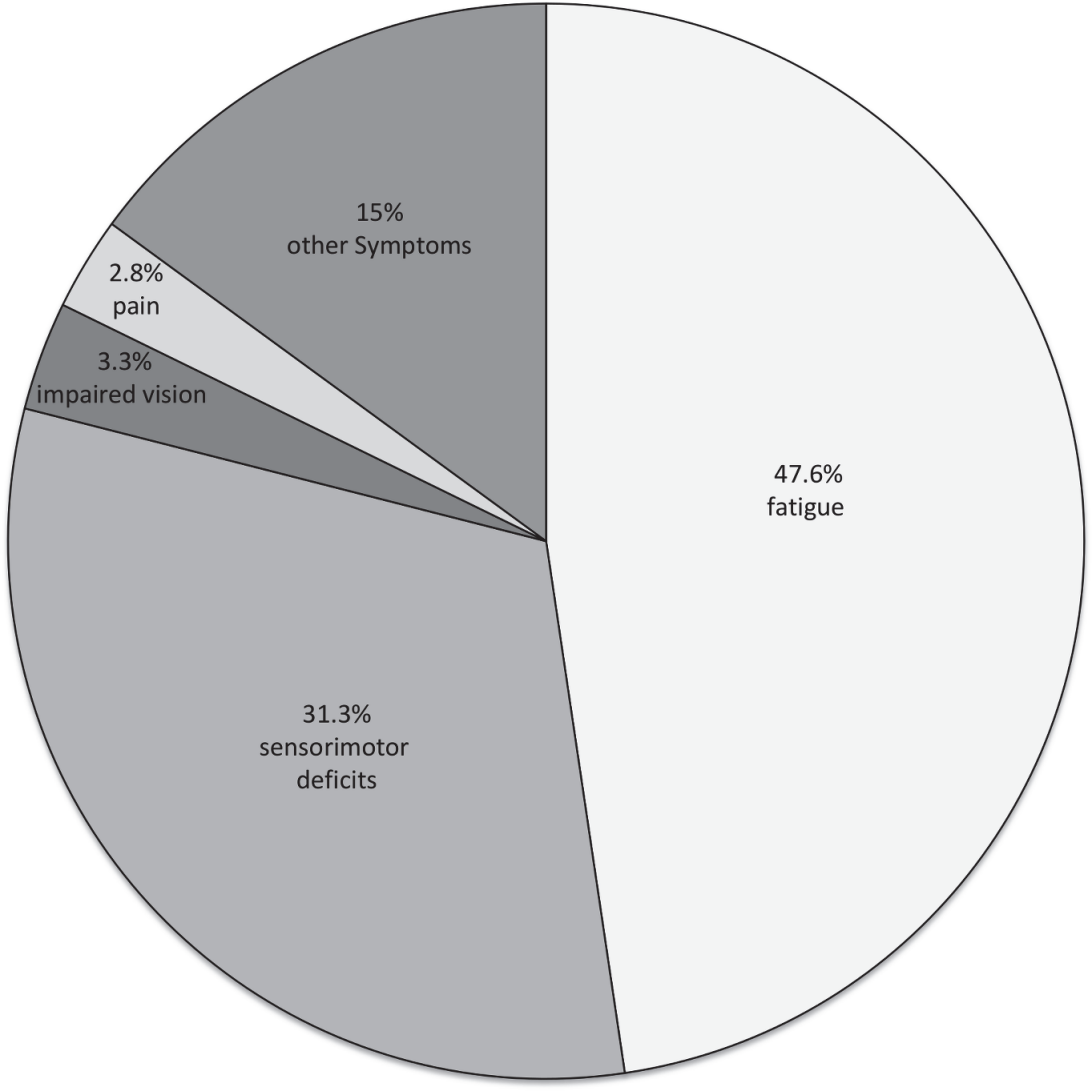
Symptoms leading to impaired working capability. In patients with reduced working capability (n = 214) fatigue is the most important reason for scaling down to part-time work or full retirement. Other symptoms: e.g. dizziness, dysarthria, bladder and bowel dysfunction, hearing loss and paroxysmal symptoms.

Overall MS was specified as the cause for reduced working capability in 214 patients (52.8%). The main reason for MS-related altered working capability was fatigue. Other reasons are shown in Fig 1.

### Working capability in relation to disability

Minimal disability (EDSS 0–2.5) was scored in 175 patients, whereas 149 patients had moderate disability (EDSS 3.0–4.5) and 81 patients had severe disability (EDSS 5.0 and higher). Patients with higher disability had significantly higher age and disease duration. For demographic details see Table 3.

**Table 3:** Demographics in different stages of disability.

	Minimal disability (0–2.5)	Moderate disability (3.0–4.5)	Severe disability (5.0–10)
**N (%)**	175 (43.2)	149 (36.8)	81 (20.0)
**Female (%)**	128 (73.1)	85 (57.0)	53 (65.4)
**Mean age (SD)**	40.1 (9.8)	46.0 (9.0)*	49.9 (9.4)* ^+^
**Mean disease duration (SD)**	8.8 (5.9)	13.6 (8.5)*	17.7 (8.8)* ^+^

*p<0.001 compared to patients with minimal disability

^+^p = 0.001 compared to patients with moderate disability

**Fig 2:**
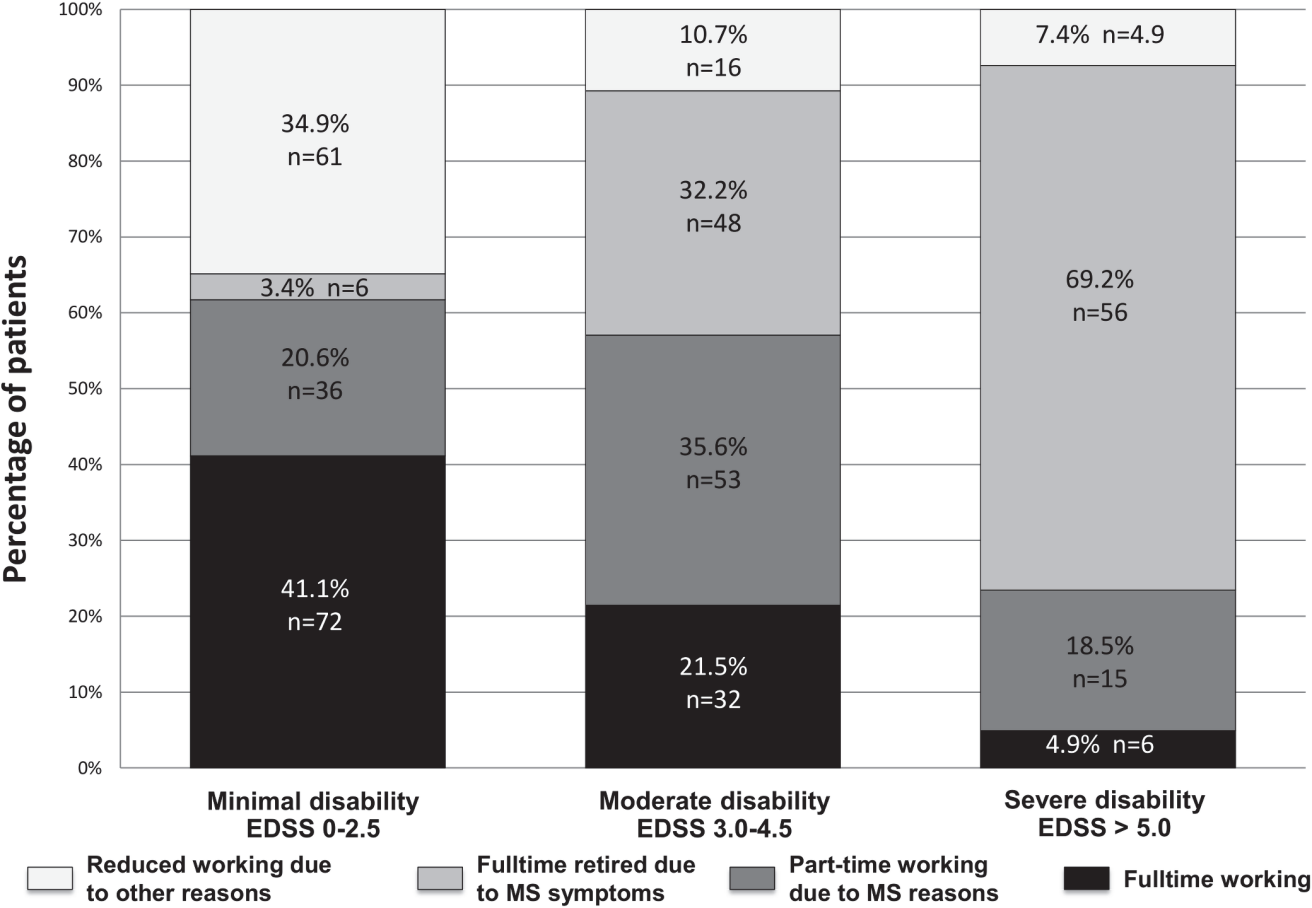
Working capability in relation to the degree of disability. With increasing disability the proportion of fulltime working patients is decreasing. Furthermore, in patients with moderate and severe disability the proportion of patients who are not employed due to other reasons than MS symptoms is lower than in the general population. One reason could be that patients with higher disability are more likely to be eligible for early retirement and are not available in the labour market.

In the group of minimal disabled 41.1% of the patients were fulltime employed, 20.6% worked part-time due to MS reasons and 3.4% were not able to work. 34.9% were not employed due to other reasons than MS, mostly care for children or household. The proportion of fulltime-employed was 21.5% in the group of patients with moderate disability and decreases to 4.9% in the group of patients with severe disability. Detailed data and the changing of working capability in relation to disability are shown in Fig 2.

## Discussion

In the present study we aimed to determine employment status and predictors for loss of working capability in a Swiss cohort of MS patients. This is the first work dealing with data about working capability in a cohort of Swiss MS patients in a real life setting. The only other analysis dealing with the Swiss population concerned members of the Swiss MS society [3]. The study is characterized by a high response rate and consistent distribution of participants over different stages of impairment [3,17,18].

We found that complete incapacity to work is lower (27%) than the previously reported for the country. One possible reason could be that our cohort contains a wide spectrum of patients at all stages of disability. The previously published data reporting a 65% rate of incapacity to work was performed among members of the Swiss multiple sclerosis society [3]. This may have included a bias for patients with higher disability and longer disease duration. Indeed, that cohort had a higher median EDSS of 5.0, a higher mean age of 52.5 years and a higher portion of patients with secondary progressive MS (SPMS) of 56.5%.

Importantly, the rate of full early retirement in our cohort is far lower than in previously reported international studies. While we found 27.1%, an international survey and studies from Australia, Western Europe and the United States revealed 40–56% of early retirement [1,2,10,19,20]. It is tempting to speculate that this lower rate in Switzerland could be the result of an exemplary system of reintegration into working life with the possibility of partial early retirement. This may also explain the low unemployment rate in our patients of 2.9% which is more or less equivalent to the unemployment rate in the general population of Switzerland [21]. As full early retirement leads to a high economic burden and lower quality of life our findings highlight the need for the possibility of partial early retirement in patients with chronic diseases [2,3,22]. Furthermore, our data are contradicting the assumption that high disability benefits are automatically leading to a higher rate of patients who don’t work as Switzerland is a country with high social standards and high disability benefits compared to other countries [4].

In our cohort disease related factors like higher EDDS, female gender and lower education level are independently associated with higher rates of incapability to work. These results are inline with previously published data from other countries [8–11]. However, other known factors for reduced employment in MS like neuropsychological dysfunction or depression were not assessed in our study [20,22].

One of the main findings of our study is that even in apparently minimally disabled patients with an EDSS of 0–2.5 24% of the patients perceive a relevant impact to the daily life in form of reduced working capability not caused by physical disability. To our knowledge data about factors leading to impaired working capability especially in minimally disabled patients are rare. Appropriately the main reason for impaired working capability in these patients was fatigue (Fig 1). This illustrates that fatigue is the major disabling symptom of MS even in minimally disabled patients and the EDSS does not provide an adequate picture of fatigue and cognitive impairment and the definition of mild disability should be reconsidered to include non-motor symptoms and daily functioning as well [7,20]. There is a need to measure and objectify fatigue and its effect on working capability. As subjective fatigue is difficult to measure new tools to quantify fatigue would be helpful. The objective measurement of alertness may offer a method of evaluating self-reported fatigue in addition to commonly used self-assessed fatigue questionnaires [23,24]. The relationship between fatigue and reduced alertness has been shown previously [23]. The inclusion of new methods to objectify fatigue could open the door for individualised treatments. In patients with a relationship between fatigue and reduced alertness wakefulness promoting stimulants like modafinil could be a therapeutic option [24]. Furthermore, treatable causes of fatigue like sleep disorders should be investigated in MS patients. The relationship between sleep disorders and fatigue and the improvement of fatigue after treating these sleep disorders has been shown previously [25,26].

Our data show that MS continues to takes its toll on the professional life of the patients early in the course. While complete incapacity becomes relevant with moderate to severe disability, many patients scale down to part-time even with minimal physical impairment. There is a need of assessing non-motor symptoms in addition to motor disability to evaluate impairment and working capability in MS patients.

## Supporting Information

S1 Source Data (PDF)
