# Supplementary material for: Variables Related to Working Capability among Swiss Patients with Multiple Sclerosis—A Cohort Study
Source: PLoS One. 2015 Apr 13;10(4):e0121856. doi: 10.1371/journal.pone.0121856 (PMC4395101; doi:10.1371/journal.pone.0121856)
Supplement: S1 Source Data — (PDF) [file pone.0121856.s001.pdf]

Patient Number	Gender 1=f 0=m	Age	Working Capabiliy 0=full 1=parttime 2=fully retired MS 3=non MS related	Disease-duration	MS Course Lublin	EDSS	Treatment yes=1	Duration between first symptoms and treatment initiation	1=University 2=Polytechnic 3=proffessional training 4=none professional training 5=other	Reason for scaling down 1=Fatigue/Cognitive 2=Paresis/Sensory/Coordination 3=Vision 4=other 5=pain
1	0	57.1	0	12.6	RR MS	4	1	7.6	3	
2	1	32.0	3	2.2	RR MS	2	1	0.8	3	
3	1	40.0	3	6.8	RR MS	2	0		3	2
4	1	54.6	0	1.4	RRMS	4.5	0		4	
5	0	40.9	0	8.6	RR MS	1.5	1	1.2	3	
6	1	27.4	1	4.8	RR MS	2	1	0.6	1	1
7	0	38.3	1	11.9	RR MS	4	1	6.3	2	1
8	1	45.5	1	21.0	RR MS	3.5	1	7.1	3	1
9	1	28.7	2	3.3	RR MS	4	1	1.1	3	1
10	1	29.1	3	8.3	RR MS	2	1	3.3	3	
11	1	46.5	2	5.1	RR MS	4	1	0.5	2	3
12	0	37.2	0	20.9	RR MS	3	1	9.9	3	
13	1	37.3	1	6.6	RR MS	3	1	0.4	3	1
14	0	58.9	1	11.4	SP MS	3	0		3	1
15	1	42.8	3	5.2	RR MS	1.5	1	0.1	3	
16	1	53.3	2	3.2	RR MS	3	1	0.5	1	1
17	1	29.7	3	10.4	RR MS	1.5	0		2	1
18	1	50.4	1	3.3	RR MS	2.5	1	0.9	3	1

19	0	60.9	0	34.0	SP MS	4	1	32.6	2	
20	1	63.0	2	27.9	SP MS	8	1	17.0	3	4
21	1	44.1	1	13.3	RR MS	1	0		2	1
22	1	25.6	0	9.5	RR MS	0	1	0.2	3	
23	0	55.1	1	3.5	PP MS	4	0		3	1
24	1	32.9	3	15.0	RR MS	1.5	1	7.7	3	
25	1	34.5	2	10.1	RR MS	4	1	3.7	3	1
26	1	41.3	3	9.2	RR MS	1.5	1	2.6	5	
27	1	53.3	3	7.7	SP MS	3	1	6.1	3	2
28	0	43.0	0	15.0	RR MS	1.5	1	6.2	3	
29	0	53.0	0	9.9	RR MS	2.5	0		3	
30	1	31.5	3	7.3	RR MS	1.5	1	0.3	3	
31	0	39.4	2	5.7	RR MS	3	1	0.5	5	1
32	1	28.3	0	1.9	RR MS	0	1	0.7	3	
33	1	53.5	2	12.4	PP MS	6	1	6.8	3	4
34	1	45.2	1	17.2	RR MS	4	1	10.8	3	2
35	1	36.5	0	8.8	RR MS	1	1	1.4	3	
36	1	28.2	1	3.7	RR MS	1.5	1	0.4	3	1
37	0	28.0	2	11.5	RR MS	6	1	2.2	3	3
38	1	42.3	2	22.8	SP MS	7.5	0		3	2
39	0	46.1	1	10.9	SP MS	4	1	2.0	3	2
40	1	33.2	1	11.7	RR MS	1.5	1	0.9	3	1
41	1	60.4	2	10.8	SP MS	6	1	5.4	2	2
42	0	42.2	0	14.9	PP MS	4	0		3	
43	1	31.2	2	10.2	RR MS	3.5	1	1.0	3	4
44	1	31.1	2	7.8	RR MS	3	1	0.2	3	1
45	0	41.7	0	26.0	PP MS	5	0		2	
46	0	61.6	1	32.4	RR MS	2	1	30.4	3	1
47	1	39.5	2	7.9	RR MS	3	1	2.1	4	1

48	1	62.9	2	18.4	SP MS	7.5	1	3.0	4	2
49	1	37.2	0	7.7	RR MS	1	1	4.4	5	
50	1	28.2	0	2.1	RR MS	0	0		1	
51	0	60.3	0	5.4	RR MS	3	1	0.8	3	
52	0	35.0	2	14.5	RR MS	4	1	0.7	2	2
53	0	39.0	0	3.9	RR MS	1.5	1	0.4	3	
54	1	54.6	3	6.3	RR MS	3.5	0		2	
55	0	38.3	0	7.9	RR MS	1.5	1	-1.0	3	
56	0	64.0	2	18.1	SP MS	4	1	9.3	1	4
57	1	46.0	3	6.2	RR MS	2.5	1	0.1	3	
58	1	59.0	3	18.4	RR MS	2.5	1	15.7	3	
59	1	40.9	1	27.0	SP MS	4.5	0		3	2
60	1	53.6	1	9.1	RR MS	5	1	3.2	3	5
61	0	47.4	2	10.3	RR MS	3	0		5	4
62	1	32.2	0	4.4	RR MS	2	0		3	
63	1	49.3	1	2.8	RR MS	2	1	0.5	2	1
64	1	44.1	2	8.4	RR MS	4	1	1.3	5	2
65	1	47.1	1	5.6	RR MS	2	1	0.2	3	2
66	1	46.8	2	17.9	SP MS	6	1	7.0	3	5
67	1	39.2	1	22.3	SP MS	6	1	9.5	1	1
68	0	46.2	0	29.9	RR MS	3.5	0		1	
69	1	34.7	3	4.5	RR MS	1.5	1	0.8	1	
70	1	32.2	1	2.9	RR MS	3	1	0.7	2	1
71	0	43.2	0	6.2	RR MS	2.5	1	0.3	2	
72	1	48.0	0	6.9	RR MS	2.5	1	3.8	3	
73	1	39.1	2	5.9	RR MS	4.5	0		3	2
74	0	24.5	0	3.3	RR MS	2	0		3	5
75	1	30.8	2	4.2	RR MS	4	1	0.5	3	4
76	1	37.4	2	3.9	RR MS	1	1	1.6	3	2

77	0	58.5	1	6.9	SP MS	6	1	0.3	3	1
78	1	33.2	1	11.3	SP MS	7	1	2.8	3	1
79	0	46.2	1	21.9	SP MS	6	1	8.8	1	2
80	0	48.0	1	3.4	PP MS	4	1	1.4	3	1
81	0	61.3	2	15.9	SP MS	6.5	1	6.2	3	1
82	1	46.0	0	10.4	RR MS	3.5	1	1.7	3	
83	1	41.0	1	13.4	RR MS	2.5	1	10.8	3	1
84	1	28.9	1	7.6	RR MS	1.5	1	0.4	3	1
85	1	43.3	0	11.0	RR MS	2	1	4.0	2	
86	1	43.5	1	13.9	RR MS	3.5	1	6.0	1	1
87	1	27.9	0	8.4	RR MS	1	1	0.3	3	
88	1	42.2	0	13.4	RR MS	1.5	1	6.2	3	
89	1	45.4	3	14.6	RR MS	1.5	1	11.3	3	
90	1	35.9	3	4.7	RR MS	2	1	1.9	3	
91	1	45.2	2	16.4	RR MS	6	1	0.0	3	1
92	1	36.8	2	7.9	RR MS	6	1	2.4	3	5
93	1	51.9	3	9.1	RR MS	1	1	1.5	3	
94	0	50.2	1	8.5	PP MS	4.5	0		3	1
95	0	36.3	3	8.0	RR MS	2	1	1.3	3	
96	1	37.5	3	5.0	RR MS	1	0		1	
97	0	29.4	2	3.5	RR MS	1.5	1	2.4	3	1
98	1	55.8	2	13.4	PP MS	6.5	0		3	2
99	1	35.3	1	6.3	RR MS	4	1	0.3	3	1
100	1	32.0	0	9.4	RR MS	3.5	1	3.8	3	
101	1	51.1	3	18.9	RR MS	2	1	10.8	3	1
102	0	32.1	2	10.3	RR MS	5	1	4.0	5	2
103	1	61.0	2	24.9	SP MS	6.5	1	13.1	5	2
104	0	49.6	0	28.4	RR MS	2	1	26.5	3	
105	1	33.6	0	8.6	RR MS	2.5	1	1.9	3	

106	0	37.3	1	8.7	RR MS	2	1	0.3		3	4
107	1	40.9	0	12.5	RR MS	3.5	1	7.1		3	
108	1	36.6	3	5.7	RR MS	1.5	1	1.3		3	
109	1	48.1	2	20.0	SP MS	6.5	1	12.0		3	1
110	0	43.9	1	20.2	SP MS	4.5	1	15.4		3	4
111	0	48.0	0	3.6	RR MS	2	1	2.1		3	
112	0	29.9	0	7.2	RR MS	1.5	1	0.4		3	
113	1	45.3	1	22.5	RR MS	4	1	20.2		3	2
114	1	38.7	3	6.3	RR MS	2	1	3.6		1	
115	1	34.5	1	11.9	RR MS	2.5	1	6.1		5	1
116	1	31.3	3	5.4	RR MS	1.5	1	4.3		3	
117	0	56.3	2	6.8	RR MS	4	1	2.5		3	1
118	0	38.7	0	14.5	RR MS	2	1	12.2		3	
119	1	35.0	2	11.0	RR MS	4.5	0			3	1
120	1	31.1	2	13.0	RR MS	3.5	1	2.2		5	1
121	0	47.7	0	25.5	RR MS	3	0			2	
122	0	40.4	0	4.9	RR MS	2	1	0.1		3	
123	1	44.0	1	3.3	RR MS	4	1	0.9		3	2
124	1	49.1	1	20.0	RR MS	2.5	0			1	1
125	0	37.6	0	14.1	RR MS	3.5	1	1.1		1	
126	0	51.7	2	7.4	RR MS	5	1	0.9		3	4
127	1	41.4	2	16.9	RR MS	6.5	0			3	2
128	1	47.3	1	19.0	SP MS	4	1	13.0		3	1
129	0	53.7	1	11.9	RR MS	2.5	1	1.3		3	1
130	1	40.5	0	9.0	RR MS	2	1	6.9		5	
131	1	49.4	2	8.3	RR MS	3.5	1	1.6		3	4
132	0	35.6	0	19.0	RR MS	1.5	1	6.9		1	
133	0	56.3	2	30.9	SP MS	7	1	20.4		3	2
134	1	28.2	2	9.9	RR MS	2	1	5.9		3	2

135	0	58.7	0	3.8	RR MS	3	1	0.5	3	
136	1	29.8	3	8.2	RR MS	1	0		3	
137	0	60.3	2	24.4	RR MS	3	0		2	1
138	0	42.6	0	9.9	RR MS	2	1	4.0	2	
139	0	41.4	1	16.6	SP MS	3	1	11.4	3	1
140	1	57.3	2	7.4	RR MS	4	1	1.1	1	1
141	1	51.1	0	19.4	RR MS	1.5	0		3	
142	1	43.3	3	13.7	RR MS	2	1	10.4	5	
143	1	44.5	3	4.0	RR MS	2	1	1.2	3	
144	1	37.4	1	15.0	RR MS	3	1	11.8	3	1
145	1	42.5	2	15.1	SP MS	6.5	0		3	4
146	1	63.0	1	33.4	RR MS	2.5	0		3	2
147	0	43.8	3	2.7	RR MS	3	1	0.9	5	
148	1	48.9	1	7.5	RR MS	2.5	1	0.6	3	1
149	1	27.0	3	7.1	RR MS	4	1	4.5	3	1
150	1	54.7	1	8.0	RR MS	2.5	1	2.4	3	4
151	0	40.0	0	6.6	RR MS	1.5	1	2.2	3	
152	1	54.5	3	24.5	RR MS	2	0		3	
153	1	52.1	1	17.4	CIS	2.5	0		3	1
154	1	45.3	1	17.2	RR MS	3	1	9.4	2	1
155	1	28.2	0	5.8	RR MS	2.5	1	0.8	2	
156	1	43.6	1	12.8	RR MS	2.5	1	7.3	3	2
157	1	52.1	1	18.0	RR MS	3	1	9.0	1	2
158	0	52.1	2	18.2	SP MS	6	1	6.3	3	2
159	1	39.6	0	4.4	RR MS	1.5	1	3.5	1	
160	0	58.0	2	16.6	SP MS	4	1	3.9	3	1
161	1	58.0	2	10.5	RR MS	4	1	4.6	3	2
162	0	55.0	0	15.9	SP MS	4	1	9.9	3	
163	1	47.8	3	20.9	RR MS	3	1	18.3	3	

164	1	54.9	2	29.4	RR MS	4	1	27.6	3	4
165	0	43.4	1	15.0	RR MS	4	1	6.5	2	2
166	1	49.3	2	28.0	SP MS	4.5	1	18.4	3	1
167	1	59.1	1	15.1	SP MS	1.5	1	1.6	1	4
168	0	22.7	0	4.5	RRMS	1	1	1.1	3	
169	1	26.9	0	3.5	RR MS	2	1	2.2	2	
170	0	58.3	3	29.8	RR MS	3	1	26.5	3	2
171	1	42.2	2	5.8	RR MS	3.5	1	0.4	4	2
172	0	39.8	1	8.5	RR MS	3.5	1	0.6	2	1
173	1	55.8	3	32.4	SP MS	3.5	1	30.4	3	
174	0	60.2	0	9.0	PP MS	3.5	0		2	
175	0	28.7	1	8.0	RR MS	5	1	3.1	3	2
176	1	54.8	2	33.9	SP MS	5.5	1	28.3	2	2
177	1	57.5	0	37.7	SP MS	6	1	24.1	3	
178	1	50.3	1	6.0	RR MS	2.5	1	1.7	2	1
179	1	43.9	1	13.3	RR MS	2	1	0.7	3	1
180	0	19.3	0	2.9	RR MS	0	1	1.1	3	
181	1	34.5	0	11.3	RR MS	3	1	2.6	3	
182	1	31.4	3	11.4	RR MS	1.5	1	5.5	3	
183	1	55.1	3	1.9	CIS	2.5	1	0.2	3	
184	0	33.6	2	8.9	RR MS	3	1	4.2	3	1
185	0	48.7	1	5.2	SP MS	4	1	0.5	2	1
186	0	53.2	3	19.1	RR MS	4	0		2	1
187	1	55.7	2	32.0	SP MS	7	1	20.4	3	2
188	1	29.0	3	9.8	RR MS	1.5	1	0.4	3	
189	1	39.8	0	13.4	RR MS	1	0		2	
190	1	46.0	1	6.9	RR MS	3	1	3.1	3	1
191	0	47.5	1	21.9	RR MS	3	1	15.5	3	1
192	0	34.0	1	11.4	SP MS	6.5	1	1.8	3	1

193	1	31.5	3	9.0	RR MS	1.5	1	6.9	3	
194	1	59.9	3	15.4	SP MS	5.5	0		3	
195	0	36.5	0	9.8	RR MS	2	1	0.7	3	
196	1	41.4	2	12.4	RR MS	6.5	1	6.6	3	2
197	1	51.8	3	18.0	RR MS	2	1	4.0	3	
198	1	57.1	3	11.1	RR MS	1.5	1	0.7	2	
199	1	47.1	1	28.9	SP MS	7	1	16.4	3	1
200	0	33.5	0	2.7	RR MS	1.5	1	0.9	3	
201	1	45.6	1	19.9	RR MS	3	1	12.2	3	2
202	1	57.2	2	43.0	SP MS	6	1	38.2	5	2
203	1	37.1	0	9.2	SP MS	6.5	1	0.3	3	
204	1	41.3	3	12.9	RR MS	3	1	9.4	2	1
205	0	51.5	0	3.0	RRMS	2	0		1	
206	1	50.7	0	6.3	RR MS	2	0		3	
207	1	48.7	0	14.0	RR MS	1	1	11.1	2	
208	1	39.3	2	19.0	RR MS	4.5	1	15.9	5	4
209	0	58.8	0	9.4	SP MS	2.5	1	4.1	3	
210	1	47.8	1	19.6	RR MS	4	1	12.5	5	1
211	0	44.9	2	22.0	RR MS	5	1	12.3	5	2
212	1	47.5	1	4.3	RR MS	3	1	2.3	3	1
213	1	44.9	0	1.4	RR MS	1.5	1	0.2	3	
214	1	55.5	3	14.6	RR MS	1	1	6.5	2	
215	0	46.6	1	3.4	RR MS	2	1	0.4	2	1
216	1	58.4	3	17.9	PP MS	6.5	0		4	
217	0	54.8	1	31.9	SP MS	4	1	25.5	2	1
218	0	49.8	2	20.5	SP MS	7	1	6.9	3	2
219	0	39.2	2	8.0	RR MS	6	1	6.2	3	2
220	1	37.9	3	3.7	CIS	3	0		1	
221	0	34.9	0	10.6	RRMS	1.5	0		2	

222	1	61.7	1	5.3	RR MS	5.5	1	-2.6	3	2
223	1	45.2	2	19.4	SP MS	6	1	5.3	3	5
224	1	30.1	2	6.5	RR MS	5.5	1	1.1	3	1
225	1	51.3	2	12.9	SP MS	5.5	1	11.0	3	2
226	1	56.3	2	10.1	RR MS	4	1	1.1	3	3
227	1	55.5	2	35.9	SP MS	4	1	29.0	3	1
228	0	41.6	2	17.0	SP MS	6	1	15.4	5	4
229	1	34.9	2	4.9	RR MS	4	1	1.1	4	1
230	0	53.3	2	24.5	RR MS	4	1	10.7	3	1
231	1	25.1	0	4.3	RR MS	2	1	0.3	3	
232	1	54.3	1	3.9	RR MS	2.5	1	0.6	3	1
233	0	57.7	2	12.4	SP MS	5.5	1	4.5	2	1
234	1	54.0	1	5.3	RR MS	1.5	1	0.2	1	1
235	0	61.3	2	17.1	SP MS	6	1	12.9	3	1
236	1	37.4	3	4.8	RR MS	2.5	1	3.8	2	
237	1	37.0	3	10.7	RR MS	6.5	1	0.8	3	1
238	0	27.5	3	1.9	RR MS	2	1	0.3	1	
239	0	18.4	3	10.3	RR MS	2.5	1	0.5	5	
240	1	47.0	1	16.8	RR MS	2	1	8.1	3	1
241	1	44.2	1	22.0	RR MS	2.5	1	13.6	3	1
242	0	21.7	3	3.1	RR MS	1.5	1	0.3	1	
243	1	51.9	3	7.6	RR MS	2.5	0		3	
244	1	38.8	3	13.3	RR MS	2.5	1	2.5	2	
245	0	48.7	0	27.0	RR MS	4	1	24.7	3	
246	1	32.4	3	7.9	RR MS	1	1	3.2	3	
247	1	33.0	3	9.9	RR MS	2	1	9.2	3	
248	0	57.4	1	34.0	SP MS	4	1	30.3	3	1
249	1	41.6	1	15.2	RR MS	5	0		3	1
250	1	58.2	2	17.4	PP MS	7	1	6.4	5	2

251	1	48.7	2	5.7	RR MS	6.5	1	0.4	3	1
252	1	37.5	0	6.0	RR MS	3	1	2.9	3	
253	1	54.4	3	18.0	RR MS	1.5	0		5	2
254	1	53.3	2	15.3	SP MS	6	1	2.5	3	3
255	0	62.0	0	5.9	RR MS	1.5	0		3	
256	1	47.0	0	43.0	RR MS	3	1	39.9	3	
257	1	32.8	3	10.9	RR MS	2.5	1	4.8	5	
258	1	34.5	0	14.6	RR MS	2.5	0		4	
259	1	58.5	2	34.0	SP MS	6	1	28.3	5	2
260	0	34.5	0	5.1	RR MS	4.5	1	1.3	3	
261	0	46.6	1	13.9	RR MS	6	0		3	4
262	1	46.7	2	9.0	RR MS	4	1	1.1	3	4
263	1	36.0	3	5.2	RR MS	1.5	0		3	
264	0	46.0	0	18.0	RR MS	2	1	13.1	3	
265	1	41.7	0	7.7	RR MS	1	0		4	
266	0	38.6	0	18.7	SP MS	4	1	5.5	3	
267	1	35.4	1	10.3	RR MS	4	1	1.6	3	1
268	1	57.8	2	43.0	SP MS	5.5	1	33.4	5	4
269	0	44.2	0	11.3	SP MS	3.5	1	6.2	3	
270	1	45.5	0	10.2	RR MS	3	1	3.3	3	
271	0	53.7	2	16.1	SP MS	7.5	1	4.5	3	1
272	1	56.0	1	9.0	RR MS	2.5	1	7.7	3	1
273	1	31.0	1	5.8	RR MS	1	1	2.9	3	1
274	1	33.3	1	5.4	CIS	1.5	0		3	5
275	1	33.4	3	11.7	RR MS	3	0		2	
276	0	52.4	2	24.3	SP MS	6	1	16.8	3	2
277	0	43.9	0	20.4	RR MS	3	0		3	5
278	0	39.1	0	12.0	RR MS	3	1	6.0	3	2
279	1	46.7	0	6.1	RR MS	2	1	0.5	5	

280	1	44.0	3	5.8	RR MS	1	1	0.9	3	
281	0	35.0	1	2.4	RR MS	5	1	0.4	3	3
282	1	38.2	1	12.4	RR MS	3	1	5.8	3	2
283	1	34.7	1	16.2	RR MS	4	1	2.4	3	2
284	1	29.8	3	9.3	RR MS	1.5	1	0.5	3	
285	1	44.0	3	7.7	SP MS	4.5	1	0.1	3	
286	1	59.4	2	16.4	SP MS	6	1	1.5	5	4
287	0	42.1	0	10.9	RR MS	1	1	4.7	3	
288	0	26.2	1	1.7	RR MS	2.5	1	0.4	3	2
289	0	56.1	1	14.4	SP MS	4	0		3	4
290	1	60.2	2	10.3	PP MS	3.5	0		3	2
291	0	63.4	2	27.7	SP MS	7.5	1	15.3	2	2
292	1	29.2	0	8.6	RR MS	2	1	7.4	3	
293	1	53.2	1	5.7	RR MS	2.5	0		3	1
294	0	54.8	0	7.0	RR MS	3	1	2.6	3	
295	1	35.3	3	9.2	RR MS	1.5	1	1.1	3	
296	1	61.6	2	12.9	SP MS	6	1	10.2	5	4
297	1	53.6	2	26.4	SP MS	8.5	1	12.1	3	2
298	0	59.8	2	22.0	SP MS	7	1	9.8	3	4
299	0	39.1	1	13.9	RR MS	2.5	0		3	1
300	1	59.0	2	32.6	SP MS	4.5	0		3	1
301	1	35.4	3	5.1	RR MS	2.5	0		1	
302	0	47.0	0	2.0	RR MS	2	0		3	
303	0	41.9	0	11.5	RR MS	4	1	1.8	2	
304	0	32.4	0	13.0	RR MS	3	1	8.9	3	
305	1	37.5	3	11.9	RR MS	1.5	1	6.1	1	
306	0	35.7	2	15.5	RR MS	4	1	9.1	3	2
307	0	39.0	0	7.3	RR MS	2	0		3	3
308	1	33.4	2	14.3	RR MS	6.5	1	0.9	3	1

309	1	49.3	1	11.9	RR	MS	3	1	5.3	3	1
310	1	37.5	3	11.4	RR	MS	2.5	1	2.3	3	
311	0	56.5	1	9.4	RR	MS	4	1	3.2	3	1
312	1	47.0	2	28.9	SP	MS	7	1	15.0	3	2
313	1	42.5	3	1.6	PP	MS	2.5	0		3	
314	1	28.2	0	10.1	RR	MS	2	1	1.0	5	
315	0	45.5	0	5.7	RR	MS	1.5	1	1.4	3	
316	0	54.9	2	16.5	RR	MS	4.5	1	5.5	3	2
317	1	33.9	1	2.3	RR	MS	2	1	0.4	5	4
318	0	28.1	3	1.4	RR	MS	1	0		1	
319	0	51.3	1	10.9	SP	MS	4.5	1	4.6	3	4
320	1	51.1	1	13.2	RR	MS	3	1	9.2	3	5
321	1	40.5	1	10.0	RR	MS	4	0		3	4
322	1	34.1	3	7.8	RR	MS	1	1	2.3	3	
323	1	54.1	2	20.0	RR	MS	6	1	13.0	3	2
324	0	47.3	0	15.0	SP	MS	6.5	1	6.3	2	
325	1	40.6	1	11.0	RR	MS	1	1	7.7	5	1
326	1	51.0	1	21.0	RR	MS	2	1	19.4	3	1
327	1	61.2	2	28.9	SP	MS	6	1	20.5	3	1
328	1	55.2	3	12.9	SP	MS	6	1	7.4	3	2
329	1	54.6	1	10.7	RR	MS	4.5	1	2.3	3	1
330	1	55.3	3	7.4	PP	MS	6	0		3	
331	1	47.1	3	1.5	RR	MS	1	1	0.2	3	
332	0	44.2	1	26.9	RR	MS	4	1	17.0	3	2
333	1	29.6	3	4.6	RR	MS	1	1	2.2	3	
334	1	59.6	3	6.7	RR	MS	3	1	1.8	3	1
335	1	61.0	0	2.1	RR	MS	3	1	0.6	2	3
336	1	50.1	3	19.3	RR	MS	4	1	14.8	3	
337	1	52.6	3	14.5	RR	MS	1	1	3.9	3	

338	1	50.5	2	14.3	SP MS	4	0		4	2
339	1	34.7	2	13.3	RR MS	2.5	1	8.1	3	1
340	1	43.4	3	4.1	RR MS	1.5	1	2.0	2	
341	1	42.5	0	21.4	RR MS	2	1	19.6	3	
342	1	31.2	0	2.1	RR MS	2	0		3	
343	1	30.1	0	3.4	RR MS	1.5	1	1.1	3	
344	1	46.4	2	10.2	RR MS	7.5	1	3.5	3	2
345	1	46.0	3	7.3	PP MS	4	0		5	
346	1	57.1	2	33.4	SP MS	8	0		1	4
347	0	46.0	1	7.4	RR MS	3	1	5.3	3	1
348	1	47.3	1	5.1	RR MS	3	1	1.7	3	2
349	1	51.5	0	25.0	RR MS	3.5	0		1	
350	1	29.8	0	2.0	RR MS	1.5	1	0.8	3	
351	1	36.8	1	7.9	RR MS	4	1	1.8	3	2
352	1	43.5	3	9.8	RR MS	2	1	6.4	1	1
353	1	30.8	1	4.7	RR MS	4.5	1	0.6	3	1
354	1	48.5	3	10.2	RR MS	3	1	2.1	3	2
355	1	48.9	0	4.2	PP MS	2	1	0.5	1	
356	1	41.6	2	14.4	RR MS	4	1	1.6	3	4
357	1	49.2	3	21.4	RR MS	1	1	16.2	3	
358	0	39.2	0	5.5	RR MS	3	1	0.7	3	
359	0	40.3	2	11.6	RR MS	4	1	4.6	3	2
360	1	33.6	3	4.2	RR MS	1.5	0		3	
361	1	27.9	0	7.4	RR MS	2.5	1	1.2	3	
362	0	51.2	2	18.9	SP MS	4	1	11.4	3	4
363	0	58.2	2	13.0	PP MS	6.5	0		3	2
364	0	49.0	0	19.6	RR MS	3	1	11.4	3	
365	0	42.9	0	4.8	RR MS	2	0		3	
366	1	54.1	2	27.1	RR MS	3.5	1	22.1	3	1

367	0	53.3	1	17.4	RR MS	6.5	1	11.0	3	2
368	0	44.1	2	3.6	RR MS	4	1	1.2	3	4
369	1	42.3	3	3.1	RR MS	2.5	0		3	
370	1	46.2	0	17.9	RR MS	2	1	16.7	3	1
371	0	61.1	1	7.5	RR MS	3	1	3.9	3	1
372	1	35.4	0	4.0	RR MS	1.5	1	1.4	3	
373	1	37.4	3	4.6	RR MS	2	0		3	
374	1	31.8	0	8.4	RR MS	1	0		3	
375	1	54.5	2	17.9	SP MS	4.5	1	8.1	5	1
376	1	31.3	1	23.0	RR MS	4	1	20.4	3	3
377	1	49.5	0	1.9	RR MS	1	1	0.5	1	
378	1	56.2	2	20.4	RR MS	6	1	6.3	5	4
379	0	41.7	0	5.4	RR MS	2	0		3	
380	0	63.9	2	7.2	RR MS	2.5	0		3	1
381	0	49.4	1	7.3	RR MS	4	1	1.0	3	2
382	1	28.6	1	3.3	RR MS	3	0		3	1
383	0	55.2	2	9.4	RR MS	3	1	1.7	3	2
384	1	42.9	1	5.2	RR MS	6.5	1	1.6	3	1
385	1	51.8	2	6.4	RR MS	3.5	1	4.1	3	1
386	1	58.8	3	8.7	RR MS	2	1	2.9	3	
387	1	47.8	2	24.0	SP MS	7	1	10.0	3	1
388	1	37.0	1	11.0	RR MS	3	1	4.2	3	2
389	1	35.2	2	10.0	RR MS	2	1	5.7	3	1
390	1	38.6	0	4.5	RR MS	1.5	1	0.2	3	
391	1	56.0	1	26.9	RR MS	3	1	20.3	3	1
392	1	52.3	3	11.3	RR MS	6	1	1.5	3	
393	0	25.1	0	1.0	RR MS	0	1	0.4	3	
394	1	44.5	2	10.0	SP MS	7	1	1.2	3	2
395	0	59.5	2	7.3	RR MS	4	1	1.8	2	3

396	0	29.7	0	5.3	RR MS	2	1	0.9	3	
397	1	39.4	2	22.5	SP MS	5.5	1	14.9	3	2
398	0	24.7	2	11.0	RR MS	3.5	1	1.0	3	2
399	0	30.8	0	6.9	RR MS	1	1	0.7	2	
400	0	63.1	1	11.1	SP MS	6	1	4.9	2	1
401	0	33.8	1	16.9	SP MS	4	0		3	2
402	0	56.6	2	14.1	SP MS	6	1	9.3	3	2
403	1	32.0	0	6.6	RR MS	1.5	1	1.9	3	
404	0	40.4	0	5.9	RR MS	2	1	1.9	3	
405	1	41.7	2	32.7	RR MS	3	1	23.7	2	4
